# Roles of miR-4442 in Colorectal Cancer: Predicting Early Recurrence and Regulating Epithelial-Mesenchymal Transition

**DOI:** 10.3390/genes14071414

**Published:** 2023-07-08

**Authors:** Jun Shibamoto, Tomohiro Arita, Hirotaka Konishi, Satoshi Kataoka, Hirotaka Furuke, Wataru Takaki, Jun Kiuchi, Hiroki Shimizu, Yusuke Yamamoto, Shuhei Komatsu, Atsushi Shiozaki, Yoshiaki Kuriu, Eigo Otsuji

**Affiliations:** Division of Digestive Surgery, Department of Surgery, Kyoto Prefectural University of Medicine, 465 Kajii-cho, Kawaramachi Hirokoji, Kamigyo-ku, Kyoto 602-8566, Japan

**Keywords:** biomarker, colorectal cancer, epithelial–mesenchymal transition, microRNA, recurrence

## Abstract

Early recurrence in patients with colorectal cancer (CRC) is associated with a poor prognosis. We aimed to identify circulating microRNAs that are biomarkers of early CRC recurrence and elucidate their functions. We identified miR-4442 as a candidate biomarker by microRNA array analysis comparing preoperative and postoperative plasma levels in patients with CRC, with and without recurrence. The association between preoperative plasma miR-4442 levels, clinicopathological features, and recurrence-free survival was analyzed in 108 patients with CRC after curative surgery. Furthermore, cell-function analyses were performed, and the involvement of miR-4442 in regulating epithelial–mesenchymal transition (EMT) was examined. Preoperatively plasma miR-4442 levels were associated with CRC recurrence and exhibited an incremental increase with earlier recurrence dates. Moreover, miR-4442 demonstrated high sensitivity and specificity as a potential biomarker for early CRC recurrence. The expression of miR-4442 in cancer tissues of patients with metastatic liver cancer from CRC was higher than in normal liver, CRC, and normal colorectal tissues. The overexpression of miR-4442 promoted the proliferative, migratory, and invasive activities of CRC cells, decreased levels of RBMS1 and E-cadherin, and increased levels of N-cadherin and Snail1. Plasma miR-4442 is a clinically useful biomarker for predicting the early recurrence of CRC. Furthermore, miR-4442 regulates EMT in CRC by directly targeting the messenger RNA of *RBMS1*.

## 1. Introduction

Colorectal cancer (CRC) is the third-most frequently occurring malignancy and the second-leading cause of cancer-related deaths worldwide [1]. Recurrence is a primary cause of death in patients with CRC and occurs in 10–20% of patients who have undergone curative resection [2,3,4]. Furthermore, the time to recurrence after surgery is an important prognostic factor; early recurrence in patients diagnosed with CRC has been correlated with poor prognosis [5,6], even when a cut-off point is set for recurrence [7]. Conventional tumor markers, such as carcinoembryonic antigen (CEA) and carbohydrate antigen 19-9 (CA19-9), do not have high sensitivity and specificity for predicting recurrence [8,9,10,11]. Therefore, reliable, minimally invasive, and convenient biomarkers to predict and detect recurrence, especially early recurrence, are needed to improve the prognosis for patients with CRC.

MicroRNAs (miRNAs), classified as small non-coding RNAs, are single-stranded RNA molecules 21–25 nucleotides in length that bind to target messenger RNAs (mRNA) and inhibit protein production through transcriptional interference or translational inhibition [12,13]. The relationship between cancer and miRNAs has attracted much attention in recent years [14,15,16]. In CRC, numerous circulating miRNAs have been identified as potential prognostic and diagnostic biomarkers that are better than conventional blood-based tumor markers, such as CEA and CA19-9 [17,18,19]. In addition, miRNAs have been implicated in tumor progression, chemotherapy resistance, and metastasis [20,21]; investigating the function of miRNAs can help to further understand the complex process of CRC recurrence and metastasis.

To the best of our knowledge, several circulating miRNAs have been reported as biomarkers for CRC recurrence [22,23]. Furthermore, these studies have not examined the role of miRNAs in CRC recurrence and metastasis. Thus, we aimed to identify novel circulating miRNAs with increased levels in the blood as biomarkers for the early recurrence of CRC and to elucidate their functions in CRC recurrence and metastasis.

## 2. Materials and Methods

### 2.1. Patients and Samples

In total, 108 preoperative plasma samples were collected from patients with Stage I, II, and III CRC who underwent curative resection at the Kyoto Prefectural University of Medicine Hospital, Kyoto, Japan, between January 2014 and May 2019. Preoperative plasma samples were collected on the day of surgery, and postoperative plasma samples were collected in the morning during an outpatient visit approximately one month after discharge. Postoperative plasma samples were obtained from all 52 patients without recurrence. However, postoperative plasma samples were obtained from only 17 of 56 patients with recurrence. CRC and normal colorectal tissue samples were obtained from 40 of the patients. All tumors were pathologically diagnosed as primary colorectal adenocarcinoma. In another cohort, frozen tumor tissue samples and normal liver tissue samples were collected from 20 patients with metastatic liver cancer from CRC. Preoperative plasma samples were obtained from 18 of 20 patients, and postoperative plasma samples were obtained from 10 of 18 patients. Plasma samples were collected from 50 healthy volunteers (HVs). Pathological assessment of the cancer stage was performed based on the eighth edition of the Union for International Cancer Control Tumor, nodes, and metastases staging [24], and the ninth edition of the Japanese Classification of Colorectal, Appendiceal, and Anal Carcinoma [25].

Blood samples were subjected to a three-step centrifugation protocol (1500 rpm for 30 min, 3000 rpm for 5 min, and 4500 rpm for 5 min at 4 °C) to remove residual cellular components. Plasma and frozen tissue samples were stored at −80 °C. Plasma samples used in the experiment were quality checked for hemolysis.

All procedures were conducted following the ethical guidelines set by the responsible committee on human experimentation (institutional and national) (approval No. ERB-C-1178-1) and in accordance with the Helsinki Declaration of 1964 and its later amendments. Informed consent was obtained from all patients prior to their inclusion in the study.

### 2.2. Microarray Analysis

The microarray analysis of plasma samples was performed using the 3D–Gene miRNA microarray platform (TORAY Industries, Kamakura, Japan). Of 56 patients with recurrence in this cohort, we selected the preoperative and corresponding paired postoperative plasma samples of three patients with recurrence. Similarly, of 52 patients without recurrence in this cohort, we selected the preoperative and corresponding paired postoperative plasma samples of three patients without recurrence. The selection criteria were as follows. Plasma samples of patients with recurrence were determined to have an earlier time to recurrence, whereas those of patients without recurrence were determined to have a longer observation period. All six patients (three with recurrence and three without) were pathologically diagnosed with Stage III CRC. The postoperative plasma samples were obtained before adjuvant chemotherapy. The patients had no history of preoperative treatment, postoperative complications, or any significant medical conditions. Furthermore, the patients with and without recurrence were well-matched with regard to age, primary cancer site, and pathological tumor type. Regarding the details of the microarray experiments, each 100 μL preoperative plasma sample from three CRC patients with recurrence was equally mixed to obtain 300 μL preoperative plasma samples for CRC patients with recurrence. Similarly, each 100 μL preoperative plasma sample from three CRC patients without recurrence was equally mixed to give 300 μL preoperative plasma samples for CRC patients without recurrence. Each 100 μL postoperative plasma sample from three CRC patients with recurrence was equally mixed to obtain 300 μL postoperative plasma samples for CRC patients with recurrence. Similarly, each 100 μL postoperative plasma sample from three CRC patients without recurrence was equally mixed to obtain 300 μL postoperative plasma samples for CRC patients without recurrence. Next, 2 out of 4 μL of total RNA extracted from the 300 μL plasma samples were utilized in microarray experiments. The extracted RNA was labeled with Hy5 using the Label IT miRNA Labeling Kit (Takara Bio, Kusatsu, Japan) and hybridized on a 3D–Gene chip at 32 °C for 16 h. The microarray was scanned, and images obtained were quantitated using a 3D–GeneH Scanner 3000 (TORAY Industries). The background intensity was subtracted to normalize the expression levels. Microarray images were analyzed using GenePix ProTM (Molecular Devices, San Jose, CA, USA).

### 2.3. RNA Extraction from Plasma, Tissue, and Cell Line Sample

Total RNA was extracted from 400 μL of plasma using the mirVana PARIS Kit (Ambion, Austin, TX, USA), in accordance with the instructions by the manufacturer’s protocol. The AllPrep DNA/RNA/miRNA universal kit (Qiagen, Hamburg, Germany) and miRNeasy Mini kit (Qiagen) were used to extract total RNA from frozen tissues and cell lines, respectively, in accordance with the instructions by the manufacturer’s protocol.

### 2.4. Quantitative Reverse Transcription–Polymerase Chain Reaction

Reverse transcription was performed using a High-Capacity cDNA Reverse Transcription Kit (Applied Biosystems, Foster City, CA, USA). TaqMan MicroRNA Assay (Thermo Fisher Scientific, Waltham, MA, USA) was used to detect hsa-miR-711 (Assay ID: 241090_mat), hsa-miR-4270 (Assay ID: CT47V47, custom order), and hsa-miR-4442 (Assay ID: 463327_mat). Expression levels were measured by quantitative reverse transcription–polymerase chain reaction (qRT-PCR) using a StepOnePlus PCR system (Applied Biosystems). Cycle threshold (*C*_t_) values were calculated using StepOne software (version 2.0; Applied Biosystems) with TaqMan Gene Expression Assays (Applied Biosystems), in accordance with the instructions by the manufacturer’s protocol. To normalize the data, we spiked the sample with cel-miR-39, a synthetic RNA oligonucleotide. The results were evaluated using the 2^−ΔΔCt^ method. Expression levels were normalized to that of cel-miR-39 (Assay ID: 000200) for RNA extracted from plasma samples and RNU6B (Assay ID: 001093) or β-actin (Assay ID: HS01060665_g1) for RNA extracted from frozen tissue samples and cell lines (Applied Biosystems). RBMS1 (Assay ID: Hs00249930_s1) was used as the primer set. HV plasma (HV192) and normal colorectal tissue (CRC355NT) were used as controls for plasma and tissue analysis, respectively.

### 2.5. Cell Lines and Cell Culture

The human CRC cell lines DLD-1 (CCL-221), HCT-116 (CCL-247), Caco-2 (HTB-37), and HT-29 (HTB-38) were purchased from the American Type Culture Collection in February 2022 (Manassas, VA, USA). Normal human umbilical vein endothelial cells (HUVECs; C-12203) were purchased from PromoCell in March 2020 (Heidelberg, Germany). DLD-1 cells were cultured in Roswell Park Memorial Institute-1640 medium (Nacalai Tesque, Kyoto, Japan). HCT-116 and HT-29 were cultured in McCoy’s 5A Medium (Invitrogen, Waltham, MA, USA). Caco-2 cells were cultured in Minimum Essential Medium (Nacalai Tesque). HUVECs were cultured in an endothelial cell growth medium (PromoCell). All media were supplemented with 10% fetal bovine serum (FBS; System Biosciences, Palo Alto, CA, USA), 100 U/mL penicillin, and 100 μg/mL streptomycin. CRC cells were tested and authenticated prior to use at the time of purchase according to the manufacturer’s methods and analyses, including Hoechst DNA stain method, Agar culture method, PCR-based assay, COI assay, and STR analysis. All cells were cultured in a humidified incubator at 37 °C with 5% CO_2_.

### 2.6. Transfection of miRNA

A mirVana miRNA mimic of hsa-miR-4442 (Assay ID: MC20622) and negative control (NC; negative control #1) were purchased from Thermo Fisher Scientific and individually transfected into cells at a final concentration of 3 nM using Lipofectamine RNAiMAX (Invitrogen) in accordance with the instructions by the manufacturer’s protocol.

### 2.7. Proliferation Assay

Cell proliferation was assessed using a water-soluble tetrazolium salts-8 assay with Cell Count Reagent SF (Nacalai Tesque). DLD-1 and HCT-116 cells were seeded in 24-well plates at a density of 0.5 × 10^4^ cells/well. After 24 h of incubation, cells were transfected with miR-NC or miR-4442 mimic. Cell proliferation was evaluated at 24 h intervals by measuring absorbance at 450 nm using a Thermo Scientific Multiskan FC (Thermo Fisher Scientific).

### 2.8. Migration and Invasion Assays

Transwell migration and invasion assays were performed using 24-well modified Boyden chambers (BD Biosciences, Franklin Lakes, NJ, USA). The upper surface of a 6.4 mm diameter filter with 8 μm pores was precoated with (invasion assay) or without (migration assay) Matrigel (BD Transduction). DLD-1 and HCT-116 cells transfected with miR-NC or miR-4442 mimic were seeded onto an FBS-free medium in the upper chamber at a density of 2.0 × 10^5^ and 1.0 × 10^5^ cells/well, respectively. The lower chamber contained the medium with 10% FBS. After 24 h of incubation, cells that had migrated or invaded the lower surface of the filters were fixed and stained with Diff–Quick staining reagents (Sysmex, Kobe, Japan). The stained cell nuclei in four independent fields of view were counted.

### 2.9. Prediction of Genes Targeted by miR-4442 In Silico

To predict the target genes of miR-4442, online software programs, miRDB (http://www.mirdb.org/miRDB/, accessed on 3 March 2022) and DIANA TOOLS micro T-CDS (http://www.microrna.gr/microT-CDS/, accessed on 3 March 2022), were employed.

### 2.10. Luciferase Reporter Assay

Wild-type (WT) or mutant-type (MT) 3′ untranslated region sequences of *RBMS1* mRNA were inserted into pmirGLO vectors (Promega, Madison, WI, USA), prior to performing luciferase assays. Cells were seeded in a 96-well black plate (Thermo Fisher Scientific). After 24 h of culture, 100 ng of the WT or MT vector and 3 nM of miR-NC or miR-4442 mimic were transfected into the cultured cells. The cells were incubated for 48 h at 37 °C. The assay was conducted using Dual-Luciferase Reporter Assay Kit (Promega) in accordance with the instructions by the manufacturer’s protocol. Luminous absorbance was measured using GloMax Discover Microplate Reader (Promega). Renilla luciferase activity served as the control reporter for normalization.

### 2.11. Western Blot Analysis

Anti-RBMS1 (sc-101190) antibodies were obtained from Santa Cruz Biotechnology (Dallas, TX, USA). Anti-E-cadherin (24E10), anti-N-cadherin (D4R1H), and anti-Snail1 (C15D3) antibodies were purchased from Cell Signaling Technology (Danvers, MA, USA). The anti-β-actin (A5441) antibody was purchased from Sigma-Aldrich (St. Louis, MO, USA).

Cells were lysed using the Mammalian Protein Extraction Reagent (Thermo Fisher Scientific), and supernatants were obtained by centrifugation at 14,500× *g* rpm for 5 min at 4 °C. Protein concentration was measured using the Protein Kit Wako II (Wako Pure Chemical, Osaka, Japan). Cell lysates containing 20 μg of total protein were separated by a 10% sodium dodecyl sulfate-polyacrylamide gel electrophoresis and transferred onto a polyvinylidene difluoride membrane (GE Healthcare, Chicago, IL, USA). Membranes were probed with the following primary antibodies: anti-RBMS1 (1:200), anti-E-cadherin (1:1000), anti-N-cadherin (1:1000), anti-Snail1 (1:1000), and anti-β-actin (1:20,000). Protein bands were detected using SuperSignal West Dura Extended Duration Substrate (Thermo Fisher Scientific), and images were acquired with an Amersham imager 680 (GE Healthcare). All western blot analyses were conducted in triplicate and densitometric analyses for protein quantification were performed using ImageJ software (http://rsbweb.nih.gov/ij/, accessed on 22 May 2023) (version 1.53). Band intensities were normalized to those of the corresponding β-actin bands.

### 2.12. Statistical Analysis

All statistical analyses were performed using the JMP software program (version 10) (SAS Institute, Cary, NC, USA). Continuous variables were presented as medians with interquartile ranges. Recurrence-free survival (RFS) was calculated using Kaplan–Meier analysis and differences in survival were measured using the log-rank test. Multivariate analysis was performed using the Cox proportional hazard regression model. Fisher’s exact probability test and Pearson’s χ^2^ test were used to compare the categorical variables between the two groups. The paired *t*-test and Student’s *t*-test were used to compare differences between parametric data, whereas the Wilcoxon signed-rank test and Mann–Whitney *U* test were used to compare differences between non-parametric data. All statistical tests were two-tailed, and the results with *p* < 0.05 were considered significant.

## 3. Results

### 3.1. Study Design

**Figure 1:**
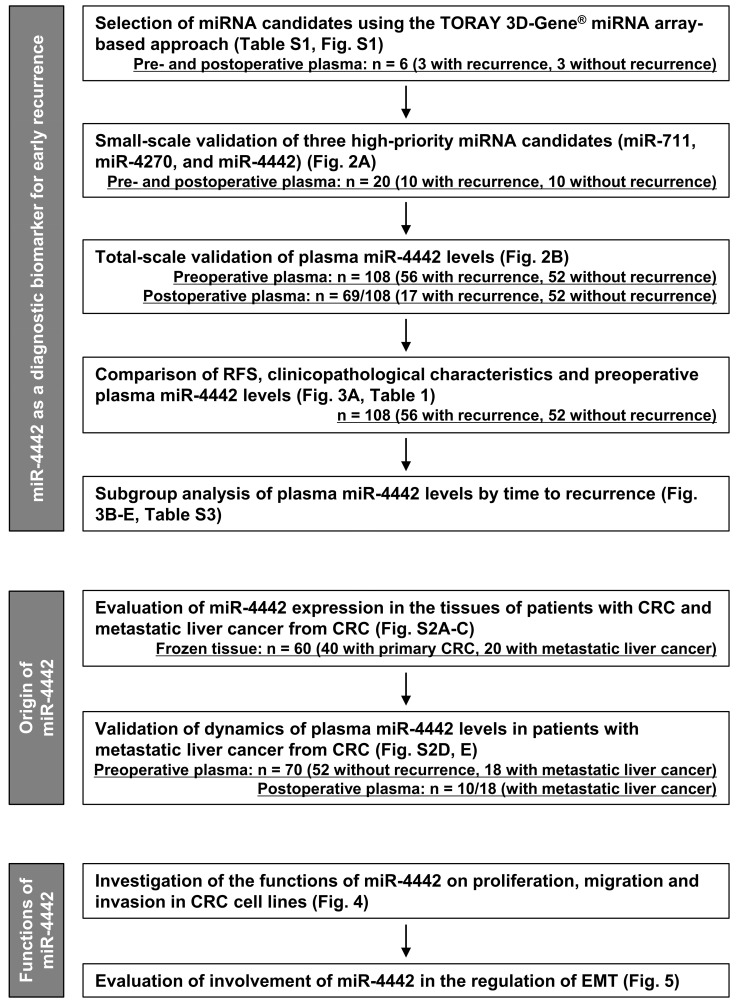
Study design. This study aimed to identify miRNAs as diagnostic biomarkers for patients with CRC with early recurrence and to determine the origin and functions of miR-4442. miRNA, microRNA; RFS, recurrence-free survival; CRC, colorectal cancer; EMT, epithelial-mesenchymal transition.

Figure 1 shows the study design. First, to identify diagnostic miRNA biomarkers for early recurrence, we conducted the following: selection of miRNA candidates using the TORAY 3D-Gene miRNA array; small-scale validation of three high-priority miRNA candidates (miR-711, miR-4270, and miR-4442); total-scale validation of plasma miR-4442 levels and analysis of RFS, clinicopathological characteristics, and preoperative plasma miR-4442 levels; and subgroup analysis of plasma miR-4442 levels by time to recurrence.

Next, we determined the origin of miR-4442 with the following methodologies: evaluation of miR-4442 expression in the tissues of patients with CRC and metastatic liver cancer from CRC; validation of the dynamics of plasma miR-4442 levels in patients with metastatic liver cancer from CRC.

Finally, we investigated the possible functions of miR-4442 in regulating the proliferative, migratory, and invasive activities of CRC cells and the involvement of miR-4442 in the regulation of epithelial–mesenchymal transition (EMT).

### 3.2. Selection of miRNA Candidates

The TORAY 3D–Gene miRNA array-based approach was used to select miRNA candidates. Figure S1 shows a heat map of representative miRNA microarray expression data from preoperative and postoperative plasma samples of CRC patients with or without recurrence. Of the 2632 miRNA candidates analyzed, 13 miRNAs that met the following criteria are listed in Table S1: the values of global normalization, which indicates the expression level of miRNA in plasma, in preoperative plasma miRNA were 200 or higher; the ratio of preoperative to postoperative plasma miRNA levels in patients with recurrence was 1.20 or lower; and the ratio of preoperative to postoperative plasma miRNA levels in patients without recurrence was 1.10 or higher. The ratio signifies the outcome obtained by dividing the preoperative and postoperative global normalization values.

We further examined miRNA candidates by using Kaplan–Meier plotter analysis (http://kmplot.com/analysis/, accessed on 22 May 2023). After referring to previous reports [26,27,28,29,30,31,32,33,34,35,36,37], miRNAs associated with cancer were given the highest priority. Overexpression of hsa-miR-4442 was associated with poor prognosis of rectal cancer patients. Thus, miR-4442 was prioritized. Based on these results, plasma levels of miR-711, miR-4270, and miR-4442 were validated in a small-scale cohort.

### 3.3. Small- and Total-Scale Validation

**Figure 2:**
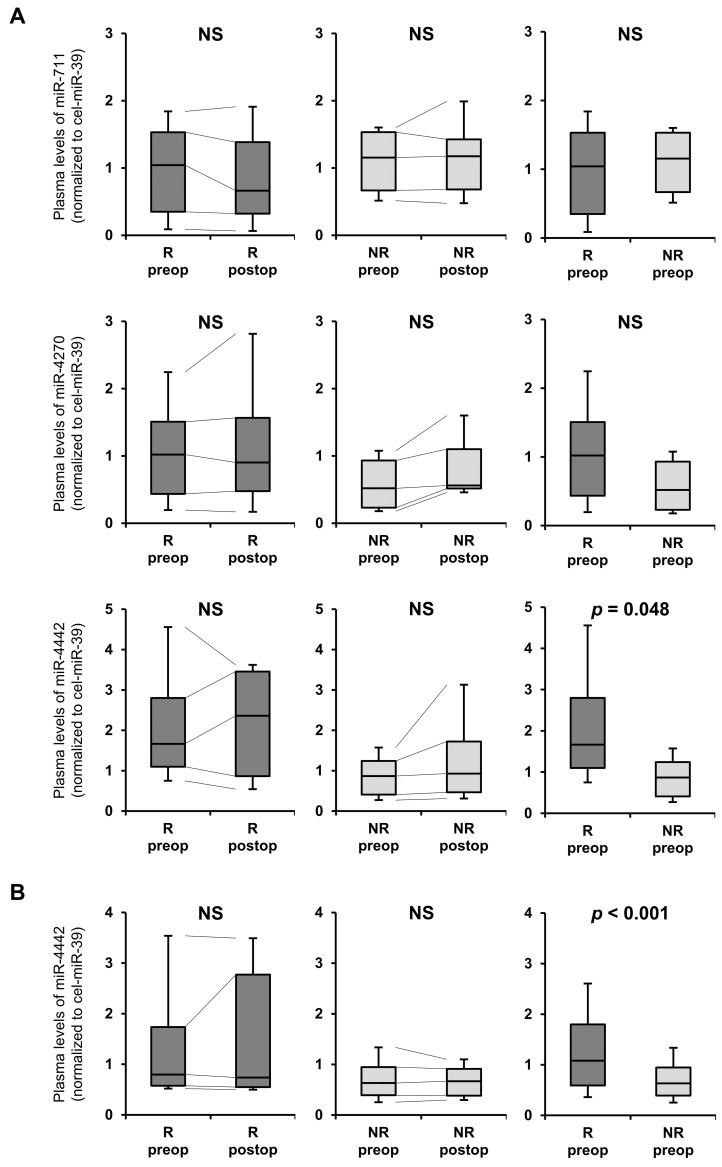
Small-scale validation of the plasma levels of miR-711, miR-4270, and miR-4442 and total-scale validation of plasma miR-4442 levels. (**A**) Small-scale validation of miR-711, miR-4270, and miR-4442 using qRT-PCR by comparing plasma levels of these miRNAs in patients with CRC with (R) and without (NR) recurrence. The dynamics of preoperative and postoperative plasma levels of miR-711, miR-4270, and miR-4442 in patients with CRC were analyzed. n = 10. (**B**) Total-scale validation of miR-4442 using qRT-PCR by comparing plasma levels in patients with CRC with (R, n = 56) and without (NR, n = 52) recurrence. The dynamics of preoperative and postoperative plasma levels of miR-4442 in patients with CRC were analyzed. The postoperative plasma samples were obtained from only 17 of 56 patients with recurrence (R), while the postoperative plasma samples were obtained from all 52 patients without recurrence (NR). (**A**,**B**) Lines between boxes indicate the medians, the outer boxes represent the 25th and 75th percentiles, and the whiskers represent the range, not including outliers. qRT-PCR, quantitative reverse transcription–polymerase chain reaction; CRC, colorectal cancer; NS, not significant; preop, preoperative plasma; postop, postoperative plasma.

In the small-scale validation, preoperative and postoperative plasma samples of patients with recurrence were determined to have an earlier time to recurrence, whereas those of patients without recurrence were determined to have a longer observation period. Figure 2A shows the results of the small-scale qRT-PCR obtained by comparing plasma miRNA levels in patients with CRC. Levels of miR-711 and miR-4270 were not significantly different between the two groups. Preoperative plasma miR-4442 levels in patients with recurrence were significantly higher than those in patients without recurrence. Furthermore, miR-4442 levels in patients with recurrence remained high after curative surgery.

In the total-scale validation, plasma miR-4442 levels in patients with recurrent CRC were significantly higher than those in patients without recurrence, and miR-4442 levels in patients with recurrence remained high after curative surgery (Figure 2B).

### 3.4. Comparison of RFS, Clinicopathological Characteristics, and Plasma miR-4442 Levels

**Figure 3:**
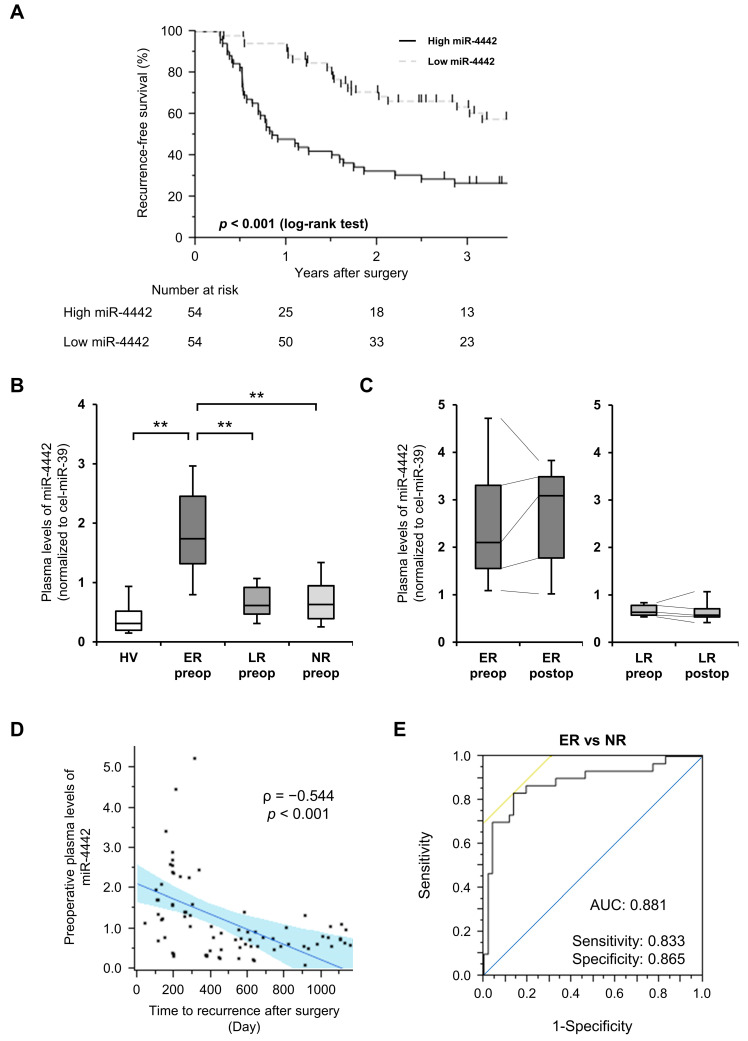
RFS of patients with CRC based on their preoperative plasma miR-4442 levels and subgroup analysis of plasma miR-4442 levels by time to recurrence. (**A**) Kaplan–Meier curves for RFS of patients with CRC based on their preoperative plasma miR-4442 levels. The differences were calculated using the log-rank test. (**B**) Comparison of plasma miR-4442 levels using qRT-PCR between HVs (n = 50) and patients with CRC with early (ER, n = 30) and late (LR, n = 26) recurrence, and without recurrence (NR, n = 52). ** *p* < 0.01. (**C**) Dynamics of preoperative and postoperative plasma miR-4442 levels using qRT-PCR in patients with CRC with early (ER, n = 8) and late (LR, n = 9) recurrence. (**D**) Correlation between preoperative plasma miR-4442 levels and time to recurrence after surgery by Spearman’s rank correlation analysis. (**E**) ROC curves and AUC values were used to evaluate the feasibility of using preoperative plasma miR-4442 levels as a diagnostic tool for early recurrence. The blue line indicates the random classifier and the yellow line indicates the iso accuracy line. (**B**,**C**) Lines between boxes indicate the medians, the outer boxes represent the 25th and 75th percentiles, and the whiskers represent the range, not including outliers. RFS, recurrence-free survival; CRC, colorectal cancer; qRT-PCR, quantitative reverse transcription-polymerase chain reaction; HVs, healthy volunteers; ROC, receiver-operating characteristic; AUC, the area-under-receiver operating characteristic curve; preop, preoperative plasma; postop, postoperative plasma.

The median miR-4442 level in the preoperative plasma of 108 patients with CRC was set as the cut-off value, and patients were placed into groups based on whether they had high or low levels. The RFS of patients with high levels was significantly worse (*p* < 0.001, Figure 3A). Multivariate analysis revealed that high preoperative plasma miR-4442 levels, venous invasion, and lymph node metastasis were independent factors for poor prognosis in patients with CRC (Table 1). Furthermore, a comparison of the characteristics and tumor factors of CRC patients with high and low preoperative plasma levels of miR-4442 showed that higher miR-4442 levels were significantly correlated with the presence of lymphatic and venous invasion, deeper tumor invasion, and lymph node metastasis (Table S2).

### 3.5. Subgroup Analysis of miR-4442 by Time to Recurrence and Diagnostic Ability of Plasma miR-4442 for Early Recurrence

As in previous studies [6,38,39,40], recurrence within one year was considered early recurrence. Recurrence after one year was considered late recurrence.

Through subgroup analysis, patients were divided into early or late recurrence groups. Clinicopathological characteristics were compared between the two groups (Table S3). However, the preoperative plasma miR-4442 levels were significantly higher in the early recurrence group than in the late recurrence group, patients without recurrence, and HVs (Figure 3B). Postoperative plasma miR-4442 levels in patients with early recurrence were significantly higher than those in patients with late recurrence and remained high after resection of the primary CRC (Figure 3C). Furthermore, time to recurrence after surgery was negatively correlated with preoperative plasma miR-4442 levels in patients with CRC, with a correlation coefficient (ρ) of −0.544, indicating a strong correlation (Figure 3D). The area under the receiver-operating characteristic (ROC) curve (AUC) value was 0.881 when comparing patients without recurrence to those with early recurrence (Figure 3E).

### 3.6. Investigation into the Origin of miR-4442

In patients with metastatic liver cancer from CRC, miR-4442 expression was significantly higher in cancerous liver tissues than in non-cancerous liver tissues. On the other hand, miR-4442 expression did not significantly vary between cancerous and normal tissues of patients with CRC (Figure S2A–C). The characteristics of patients with metastatic liver cancer are shown in Table S4.

Preoperative plasma miR-4442 levels in patients with metastatic liver cancer were significantly higher than those in patients without recurrence (Figure S2D), and these levels were significantly lower after the resection of cancerous tissue (Figure S2E).

### 3.7. Expression and Functions of miR-4442 in CRC Cell Line

**Figure 4:**
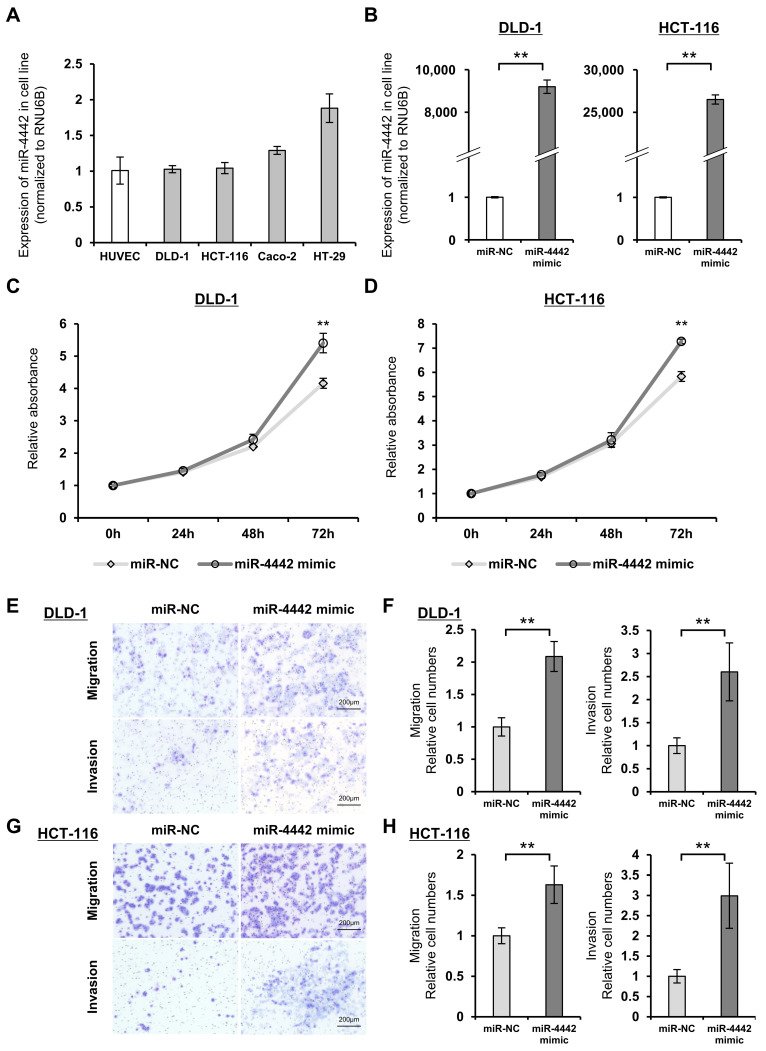
Investigation of the functions of miR-4442 in vitro. (**A**) miR-4442 expression levels measured using qRT-PCR in DLD-1, HCT-116, Caco-2, HT-29, and HUVECs. The results are presented as the mean ± standard deviation. n = 3. (**B**) miR-4442 expression levels in DLD-1 and HCT-116 cells transfected with miR-NC and miR-4442 mimics. The results are presented as the mean ± standard deviation. n = 3. ** *p* < 0.01. (**C**,**D**) Proliferation assays of DLD-1 (**C**) and HCT-116 (**D**) cells transfected with miR-NC or miR-4442 mimic. The results are presented as the mean ± standard deviation. n = 4. ** *p* < 0.01. (**E**–**H**) Migration and invasion assays of DLD-1 (**E**,**F**) and HCT-116 (**G**,**H**) cells transfected with miR-NC or miR-4442 mimic. (**E**,**G**) Representative images of transwell migration and invasion assays of DLD-1 (**E**) and HCT-116 (**G**) cells. (**F**,**H**) The mean number of cells in 12 random fields. The results are presented as the mean ± standard deviation. ** *p* < 0.01. qRT-PCR, quantitative reverse transcription–polymerase chain reaction; HUVECs, human umbilical vein endothelial cells; NC, negative control.

Referring to the results of miR-4442 expression levels measured using qRT-PCR, we decided to overexpress miR-4442 in the cell function assays and use DLD-1 and HCT-116, which possessed expression levels that were the lowest in CRC cell lines, in the assays (Figure 4A). Expression levels in these cells significantly increased following the transfection with the mimic (Figure 4B). Overexpression of miR-4442 promoted the proliferative, migratory, and invasive activities of DLD-1 and HCT-116 (Figure 4C–H).

### 3.8. Exploration of a Direct Target of miR-4442 and Evaluation of the Involvement of miR-4442 in the Regulation of EMT

**Figure 5:**
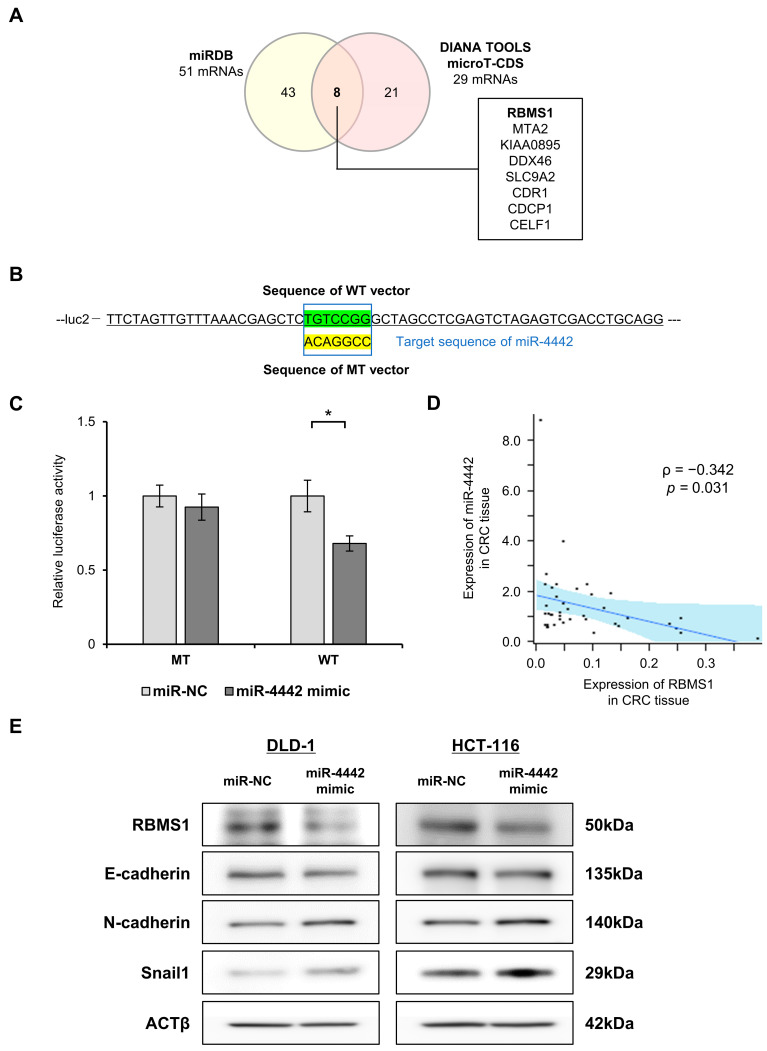
Direct target of miR-4442 and regulatory role of miR-4442 in EMT. (**A**) Downstream targets were predicted by in silico searches using two bioinformatic algorithms (miRDB and DIANA TOOLS microT-CDS). (**B**) Wild- (WT) or mutant-type (MT) sequence of *RBMS1* mRNA and sequence in the vicinity of the vector junction site. (**C**) Luciferase reporter assay in HCT-116 cells transfected with miR-NC or miR-4442 mimic. n = 3. * *p* < 0.05. (**D**) Correlation between expression of miR-4442 and *RBMS1* mRNA in CRC tissue by Spearman’s rank correlation analysis. (**E**) Western blot analyses of RBMS1 and EMT-related proteins (E-cadherin, N-cadherin, and Snail1) in DLD-1 and HCT-116 cells transfected with miR-NC or miR-4442 mimic. EMT, epithelial-mesenchymal transition; mRNA, messenger RNA; NC, negative control; CRC, colorectal cancer.

Based on these results, overexpression of miR-4442 may play a role in recurrence and metastasis by promoting migration and invasion. Therefore, we evaluated the involvement of miR-4442 in EMT regulation. By utilizing miRDB and DIANA TOOLS micro T-CDS, which are online databases for miRNA target prediction and functional annotations based on high-throughput sequencing experiments, eight genes were identified as possible miR-4442 targets (Figure 5A). KIAA0895, SLC9A2, and CDR1 have not been previously implicated in proliferation, migration, and metastasis of CRC. Conversely, MTA2, DDX46, CDCP1, and CELF1, which have been reported to play a role in at least one of these processes in CRC, are known to promote cancer [41,42,43,44]. This contradicts the findings of this study. On the other hand, in a previous study, RBMS1 has been shown to regulate the EMT and metastasis of CRC [45]. Additionally, the overexpression of miR-4442 resulted in a reduction in luciferase activity in the vector harboring the target sequence of *RBMS1* mRNA (wild-type vector) while not affecting the vector containing the mutant sequence (mutant-type vector) (Figure 5C). CRC tissue samples obtained from 40 patients were used to analyze the expression of miR-4442 and *RBMS1* mRNA. Expression of miR-4442 in cancer tissue was negatively correlated with the expression of *RBMS1* mRNA in cancer tissue, with a correlation coefficient (ρ) of −0.342 (Figure 5D). Hence, these findings indicate *RBMS1* mRNA as a direct target gene of miR-4442. The correlation between miR-4442 levels, RBMS1, and EMT-related proteins, such as E-cadherin, N-cadherin, and Snail1, was investigated in DLD-1 and HCT-116 cells transfected with the mimic using Western blotting analysis. Overexpression of miR-4442 decreased levels of RBMS1 and E-cadherin and increased those of N-cadherin and Snail1 (Figure 5E and Figure S3).

## 4. Discussion

In the present study, plasma miR-4442 was identified as a diagnostic preoperative miRNA biomarker for early recurrence in patients with CRC. Furthermore, the expression of miR-4442 in cancerous tissues of patients with metastatic liver cancer from CRC was higher than that in normal liver, CRC, and normal colorectal tissues. The overexpression of miR-4442 promoted the proliferative, migratory, and invasive activities of CRC cells, and miR-4442 regulated EMT in CRC by directly targeting *RBMS1* mRNA. Based on the results of this study, the molecular mechanisms associated with miR-4442 and the possibility of its clinical application in patients with CRC are discussed below.

The plasma miR-4442 levels in patients with CRC, especially those with recurrence, were higher than those in HVs. In order to investigate the origin of miR-4442, we conducted an analysis of its expression levels across tissues. Initially, we hypothesized that CRC tissue would serve as the origin and, thus, exhibit high expression levels of miR-4442. However, the obtained results were in contrast to our expectations. Consequently, our focus shifted to investigating miR-4442 expression in liver metastatic tissue. Notably, we observed a significant upregulation of miR-4442 in the cancerous regions of liver metastases. miRNA levels in plasma are not necessarily representative of miRNA expression in cancer cell lines and tissues [46,47]. However, in this study, miR-4442 expression was high in metastatic liver cancer tissues but decreased after resection. In contrast, plasma miR-4442 levels did not decrease after curative resection of primary CRC tissue. These findings suggest that miR-4442 is heterogeneously overexpressed in CRC tissue, and that some CRC cells overexpressing miR-4442 are associated with migration, invasion of endothelial vascular cells, formation of liver metastasis, and proliferation in liver metastatic lesions. As a result, a high level of miR-4442 in the preoperative plasma of patients with CRC might micrometastasis for lymph nodes and/or distant organs such as liver metastasis.

Several aspects of the molecular functions of miR-4442 remain unclear. Previous studies have indicated that miR-4442, which targets CD6, may be involved in Grave’s disease and Hashimoto’s thyroiditis [48] and in the proliferation or apoptosis in small airway epithelial cells exposed to transforming growth factor-β [49]. However, there have been no detailed reports on the regulatory function of miR-4442 and its involvement in cancer; therefore, we consider our findings to be significant. The present study suggests that miR-4442 regulates cell proliferation, migration, and invasion in CRC cells, which could be explained by inhibiting the function of one of its target genes, the cancer-suppressor gene *RBMS1*. A previous study has demonstrated that RBMS1 suppressed cell proliferation, albeit in prostate cancer [50]. RBMS1 has been implicated in cell cycle regulation through its interaction with the upstream region of the *MYC* gene [51], which plays an important role in DNA replication and gene transcription. Therefore, miR-4442 and RBMS1 may regulate cell proliferation through a similar mechanism in CRC cell lines. In addition, it is suggested that the roles and functions of miR-4442 and RBMS1 may vary across different subtypes of CRC. Considering the diverse treatment strategies, such as chemotherapy, employed for distinct CRC subtypes, the characterization of miR-4442 and RBMS1 in relation to these subtype differences is of considerable interest. If RBMS1 is indeed associated with the upstream region of the *MYC* gene, miR-4442 and RBMS1 could potentially play a role in the canonical type of CRC. Regrettably, there is a dearth of reports addressing this particular information, necessitating further validation. Although not demonstrated in this study, it is speculated that miR-4442 may be implicated in the development of drug resistance in CRC. CRC cells overexpressing miR-4442 could potentially acquire resistance to chemotherapy, thereby potentially contributing to metastasis and recurrence. Furthermore, miR-4442 regulated EMT through RBMS1, a suppressor of mesenchymal characteristics in colon cancer cells [45]. In particular, AKAP12, one of the direct targets of RBMS1, is involved in protein kinase A- and C-signaling cascades and is a negative regulator of Snail1 [45,52]. Snail1 binds to three E-boxes of the E-cadherin gene (*CDH1*) promoter and represses transcription, causing epithelial cells to acquire a migratory mesenchymal phenotype [53]. Therefore, miR-4442 may play a role in the onset of EMT in CRC metastasis by regulating the RBMS1-AKAP12-Snail1-E-cadherin axis.

Considering the clinical application of this study, plasma miR-4442 could be a preoperative predictive biomarker, especially for early recurrence, in CRC patients. Close outpatient observation of patients with high preoperative plasma miR-4442 levels will lead to early detection of recurrence, which will ensure prompt treatment with adjuvant chemotherapy and curative resection, ultimately improving the prognosis for patients [54]. Furthermore, targeted treatment, such as the inhibition of miR-4442 expression, may prevent CRC recurrence by suppressing EMT. Thus, miR-4442 may have implications for the prognosis improvement of CRC patients.

The present study has several limitations. First, the sample size of the cohort was relatively small. Tissue samples from patients could not be verified within the same cohort because of the difficulty of collecting more samples. Second, the cut-off values for the plasma miR-4442 levels were derived from the results of the current study, no previous studies. Third, this study may have misclassified some patients as recurrence-free due to the short observation period. To address these limitations, the preliminary findings of this study need to be validated in a larger cohort and many regions over a longer observation period.

## 5. Conclusions

In the study, miR-4442 was identified as a diagnostic blood-based biomarker for recurrence, especially early recurrence, in patients with CRC, and the expression of miR-4442 was high in the cancer tissues of patients with metastatic liver cancer from CRC. Furthermore, miR-4442 increased the malignant potential of CRC cells and was involved in EMT regulation in CRC cells. Our findings provide novel insights into CRC treatment, including that patients with high preoperative plasma miR-4442 levels should be closely monitored and that clinical drugs targeting miR-4442 may improve the prognosis of patients with CRC by suppressing CRC recurrence and metastasis.

## Figures and Tables

**Table 1:** Univariate and multivariate analyses for recurrence-free survival rates of patients with colorectal cancer.

			Univariate	Multivariate		
Variable	Total (n = 108)	3 yrs RFS (%)	*p* Value	*p* Value	HR	(95% CI)
Age (year)						
≥70	42	42.8	0.664			
<70	66	46.4				
Sex						
Male	51	45.5	0.909			
Female	57	44.6				
Neoadjuvant chemotherapy						
Present	12	38.9	0.782			
Absent	96	45.9				
Adjuvant chemotherapy						
Present	45	33.7	0.182			
Absent	63	52.2				
Tumor location						
Colon	55	44.9	0.408			
Rectum	53	43.3				
Tumor type ^†^						
Differentiated type	99	47.2	0.066			
Undifferentiated type	9	22.2				
Tumor size^†^ (mm)						
≥40	54	30.4	0.016	0.271	1.404	0.771–2.630
<40	54	59.5				
Lymphatic invasion ^†‡^						
Present	61	38.3	0.043	0.788	1.087	0.583–1.982
Absent	47	53.8				
Venous invasion ^†‡^						
Present	54	23.7	<0.001	0.024	2.160	1.103–4.419
Absent	54	67.9				
Tumor depth ^†§^						
T3/4	78	34.9	0.002	0.867	1.077	0.461–2.675
T1/2	30	65.5				
Lymph node metastasis ^†§^						
Positive	56	24.5	<0.001	0.026	1.931	1.080–3.600
Negative	52	66.7				
CEA (ng/mL)						
≥5	54	31.4	0.006	0.528	1.205	0.679–2.178
<5	54	57.7				
CA19-9 (U/mL)						
≥37	12	8.3	<0.001	0.097	1.927	0.883–3.934
<37	96	50.1				
Preoperative plasma level of miR-4442						
High (≥0.79)	54	26.8	<0.001	0.012	2.074	1.175–3.760
Low (<0.79)	54	63.7				

RFS recurrence-free survival, HR hazard ratio, CI confidence interval, CEA carcinoembryonic antigen, CA19-9 carbohydrate antigen 19-9. ^†^ Pathological diagnosis. ^‡^ According to the 9th edition of the Japanese Classification of Colorectal, Appendiceal, and Anal Carcinoma. ^§^ According to the 8th edition of the International Union Against Cancer tumor, node, metastasis classification system.

## Data Availability

The data presented in this study are available on request from the corresponding author.

## Supplementary Materials

The following supporting information can be downloaded at: https://www.mdpi.com/article/10.3390/genes14071414/s1, Figure S1: A heat map of representative miRNA microarray expression data from preoperative and postoperative plasma samples of CRC patients with or without recurrence; Figure S2: Expression of miR-4442 in tissues, dynamics of plasma miR-4442 levels in patients with metastatic liver cancer from CRC; Figure S3: Densitometric analyses for protein quantification were performed using ImageJ software (version 1.53); Table S1: Comparison of preoperative and postoperative plasma microRNA levels in colorectal cancer patients with or without recurrence; Table S2: Comparison of characteristics and tumor factors of colorectal cancer patients with high and low preoperative plasma levels of miR-4442; Table S3: Comparison of clinicopathological characteristics of colorectal cancer patients with early and late recurrence; Table S4: Characteristics of patients with metastatic liver cancer from colorectal cancer.
