# Supplementary material for: Roles of miR-4442 in Colorectal Cancer: Predicting Early Recurrence and Regulating Epithelial-Mesenchymal Transition
Source: Genes (Basel). 2023 Jul 8;14(7):1414. doi: 10.3390/genes14071414 (PMC10378884; doi:10.3390/genes14071414)
Supplement: Supplementary file 1 [file genes-14-01414-s001.zip › Table S1.pdf]

Table S1. Comparison of pre- and postoperative plasma microRNA levels in colorectal cancer patients with or without recurrence

	Global normalization				Ratio	
Name	R preop	R postop	NR preop	NR postop	R preop/R postop	NR preop/NR postop
hsa-miR-4442	921	782	710	557	1.18	1.27
hsa-miR-4270	307	311	286	209	0.99	1.37
hsa-miR-711	255	249	213	179	1.02	1.19
hsa-miR-4675	290	242	251	159	1.2	1.58
hsa-miR-6802-5p	476	399	409	315	1.19	1.3
hsa-miR-6858-5p	445	426	401	328	1.04	1.22
hsa-miR-4689	988	827	705	577	1.19	1.22
hsa-miR-6771-5p	706	715	696	591	0.99	1.18
hsa-miR-6721-5p	315	263	213	182	1.2	1.17
hsa-miR-6756-5p	545	522	492	423	1.05	1.17
hsa-miR-10400-5p	34746	30582	24138	20788	1.14	1.16
hsa-miR-6738-5p	312	320	282	246	0.98	1.15
hsa-miR-3141	317	274	246	217	1.16	1.13

R preop: preoperative plasma of colorectal patients with recurrence

R postop: postoperative plasma of colorectal patients with recurrence

NR preop: preoperative plasma of colorectal patients without recurrence

NR postop: postoperative plasma of colorectal patients without recurrence
