# Supplementary material for: Roles of miR-4442 in Colorectal Cancer: Predicting Early Recurrence and Regulating Epithelial-Mesenchymal Transition
Source: Genes (Basel). 2023 Jul 8;14(7):1414. doi: 10.3390/genes14071414 (PMC10378884; doi:10.3390/genes14071414)
Supplement: Supplementary file 1 [file genes-14-01414-s001.zip › Table S2.pdf]

Table S2. Comparison of characteristics and tumor factors of colorectal cancer patients with high and low preoperative plasma levels of miR-4442

Variable	Preoperative plasma level of miR-4442 ≥ 0.79 (n = 54)	Preoperative plasma level of miR-4442 < 0.79 (n = 54)	*p* value
Age (year), median (IQR)	69 (62–77)	68 (61–71)	0.243
Sex			
Male	23	28	0.335
Female	31	26	
Neoadjuvant chemotherapy			
Present	5	7	0.540
Absent	49	47	
Adjuvant chemotherapy			
Present	26	19	0.172
Absent	28	35	
Tumor location			
Colon	26	29	0.564
Rectum	28	25	
Tumor type[†]			
Differentiated type	48	51	0.296
Undifferentiated type	6	3	
Tumor size[†] (mm), median (IQR)	39 (23–55)	39 (22–57)	0.953
Lymphatic invasion[†‡]			
Present	38	23	0.004
Absent	16	31	

Venous invasion[†‡]			
Present	36	18	< 0.001
Absent	18	36	
Tumor depth[†§]			
T3/4	44	34	0.032
T1/2	10	20	
Lymph node metastasis[†§]			
Positive	36	20	0.002
Negative	18	34	
Stage[†§]			
I	5	15	0.005
II	13	19	
III	36	20	
CEA (ng/mL)	6.2 (2.7–11.4)	4.5 (2.3–8.1)	0.238
CA19-9 (U/mL)	9.0 (3.3–25.4)	6.4 (3.4–15.1)	0.349

IQR interquartile range, *CEA* carcinoembryonic antigen, *CA19-9* carbohydrate antigen 19-9

[†] Pathological diagnosis

[‡] According to the 9th edition of the Japanese Classification of Colorectal, Appendiceal, and Anal Carcinoma

[§] According to the 8th edition of the International Union Against Cancer tumor, node, metastasis classification system
