# Supplementary material for: Roles of miR-4442 in Colorectal Cancer: Predicting Early Recurrence and Regulating Epithelial-Mesenchymal Transition
Source: Genes (Basel). 2023 Jul 8;14(7):1414. doi: 10.3390/genes14071414 (PMC10378884; doi:10.3390/genes14071414)
Supplement: Supplementary file 1 [file genes-14-01414-s001.zip › Table S3.pdf]

Table S3. Comparison of clinicopathological characteristics of colorectal cancer patients with early and late recurrence

Variable	Early recurrence (n = 30)	Late recurrence (n = 26)	*p* value
Age (year), median (IQR)	71 (61–79)	64 (60–70)	0.059
Sex			
Male	13	14	0.432
Female	17	12	
Neoadjuvant chemotherapy			
Present	3	4	0.543
Absent	27	22	
Adjuvant chemotherapy			
Present	13	14	0.432
Absent	17	12	
Tumor location			
Colon	14	12	0.969
Rectum	16	14	
Tumor type[†]			
Differentiated type	26	23	0.840
Undifferentiated type	4	3	
Tumor size[†] (mm), median (IQR)	40 (26–59)	51 (39–62)	0.130

Lymphatic invasion†‡			
Present	22	14	0.129
Absent	8	12	
Venous invasion†‡			
Present	24	16	0.127
Absent	6	10	
Tumor depth†§			
T3/4	26	23	0.840
T1/2	4	3	
Lymph node metastasis†§			
Positive	22	17	0.519
Negative	8	9	
Stage†§			
I	1	2	0.708
II	7	7	
III	22	17	
CEA (ng/mL)	6.5 (3.2–14.7)	6.4 (3.1–12.0)	0.407
CA19-9 (U/mL)	10.5 (4.1–41.1)	10.3 (3.3–26.2)	0.748
Recurrence pattern			

Hematogenous	26	17	0.060
Other than hematogeneous	4	9	
Preoperative plasma level of miR-4442, median (IQR)	1.74 (1.29–2.50)	0.61 (0.44–0.93)	< 0.001

IQR interquartile range, *CEA* carcinoembryonic antigen, *CA19-9* carbohydrate antigen 19-9

† Pathological diagnosis

‡ According to the 9th edition of the Japanese Classification of Colorectal, Appendiceal, and Anal Carcinoma

§ According to the 8th edition of the International Union Against Cancer tumor, node, metastasis classification system
