# Supplementary material for: Roles of miR-4442 in Colorectal Cancer: Predicting Early Recurrence and Regulating Epithelial-Mesenchymal Transition
Source: Genes (Basel). 2023 Jul 8;14(7):1414. doi: 10.3390/genes14071414 (PMC10378884; doi:10.3390/genes14071414)
Supplement: Supplementary file 1 [file genes-14-01414-s001.zip › Table S4.pdf]

Table S4. Characteristics of patients with metastatic liver cancer from colorectal cancer

Variable	Total (n = 20)
Age (year), median (IQR)	69 (49–76)
Sex	
Male	10
Female	10
Neoadjuvant chemotherapy	
Present	13
Absent	7
Adjuvant chemotherapy	
Present	7
Absent	13
Tumor location	
Colon	14
Rectum	6
Tumor type of primary colorectal†	
Differentiated type	20
Undifferentiated type	0
Metastasis	
Synchronous	5
Metachronous	15

IQR interquartile range

† Pathological diagnosis
